# Multimedia Tilt Photography-Assisted Remote Sensing Technology in Mine Ecological Restoration

**DOI:** 10.1155/2022/1442738

**Published:** 2022-06-10

**Authors:** Yanjun He, Shuzhao Chen, Bin Zhang, Kai Chen

**Affiliations:** ^1^Lijiahao Coal Mine, National Energy Baotou Energy Co., Ltd, Ordos, Inner Mongolia Autonomous Region 017000, China; ^2^School of Mines, China University of Mining and Technology, Xuzhou, Jiangsu 221116, China

## Abstract

Ecological restoration with the assistance of certain artificial measures is to restore the original ecological function and productivity of the ecosystem or to make the ecosystem develop to a virtuous circle, which is a complex systematic project. Based on this, combined with the actual needs of a mine ecological restoration project, this paper puts forward the technical process and method of quickly acquiring the geographic information of abandoned mines by using the oblique photography technology of unmanned aerial vehicles. The verification results show that the maximum plane/elevation residual error of the checkpoints measured in the field is 0.221 m−0.181 m, and the median error is 0.030 m–0.112 m. According to the encryption requirement of 1∶500 scale topographic mapping in hilly land, the plane position error of the inspection point relative to the field control point should be less than 0.175 m. The elevation should be less than 0.280 m, and the experimental results in this paper can meet the requirements. Oblique photography can provide abundant digital results, and it can play an important role in mine restoration scheme design, restoration construction, and monitoring and management after restoration.

## 1. Introduction

China is one of the countries with the most serious soil erosion in the world today. Soil erosion leads to a series of problems such as land degradation, sediment deposition, and ecological deterioration, which poses a serious threat to the survival and development of human society [1]. Soil and water conservation monitoring is aimed at soil erosion and its control. It collects information of soil erosion factors (model parameters), types (including precipitation), intensity, and treatment status (measures and benefits) by means of surface observation, investigation, remote sensing interpretation, and simulation calculation at small watershed and regional scale [2]. Traditional monitoring methods mainly adopt manual investigation, which is time-consuming and laborious, and can only monitor the soil erosion in a small range. It is difficult to quantitatively monitor a large area. Using remote sensing technology to monitor soil and water conservation can overcome the defects of traditional monitoring methods. By interpreting remote sensing images, the distribution map of soil erosion factors, types, and erosion intensity can be made, thus achieving the purpose of monitoring soil and water conservation, and it has become the main method for monitoring soil and water conservation in large areas at present [3]. The organic combination of high-resolution remote sensing images and unmanned aerial vehicle images can effectively observe and sample the observed objects with different scales of soil and water conservation and can grasp the dynamic monitoring of soil and water loss from a macro perspective [4]. Inclination photogrammetry is a new and high technology developed in surveying and mapping field in recent years. It overturns the limitation that photogrammetry technology must shoot from vertical angle in the past. By carrying multiple sensors on the same flight platform and collecting images from one vertical and multiple different angles at the same time, through a series of internal work such as image recognition, solution, and mapping, a true 3D model is established, and the real world can be quickly restored, allowing users to acquire images from multiple angles. In view of this research problem, China's remote sensing monitoring of mines has also developed from the ground remote sensing platform to the aviation and aerospace remote sensing platform, and the monitoring data have also changed from the initial thermal infrared data to the medium-low resolution data and then to the high-resolution remote sensing image data monitoring [5]. Yutaka et al. put forward the thermal infrared radiation of coal seam by taking coal mine as an experiment and investigated Coalfield investigation, prediction, coal prospecting, and geological hazard detection [6]. Cui et al. monitored the landslide by unmanned aerial vehicle, drew orthographic mosaic DOM and DTM, and evaluated the landslide surface [7]. Szade and others thought that UAV was an unmanned aircraft operated by radio remote control equipment and self-provided program control device. Compared with traditional mine monitoring technology, in addition to the advantages of high resolution, strong pertinence, small size, convenient use, and low cost, UAV was also an effective supplement to traditional aerial photogrammetry. At the same time, UAV technology is characterized by automation, intelligence, and specialization. UAV had flexible data acquisition and strong maneuverability. The research of UAV tilt photogrammetry had been discussed by scholars from different angles. On the basis of current research, combined with the actual needs of a mine ecological restoration project, this paper puts forward the technical process and method for quickly acquiring the geographic information of abandoned mines by using the oblique photography technology of unmanned aerial vehicles [8]. The verification results show that the oblique photography technology can provide richer digital results, and it can play an important role in mine restoration scheme design, restoration construction, and monitoring and management after restoration.

## 2. Methods

### 2.1. Technical Planning and Data Processing of Tilt Photography in Test Area

#### 2.1.1. Geomorphological Survey of the Test Area

The experimental area is located in the mountainous area of Loess Plateau in northwestern Shanxi, with rugged terrain and many gullies. Hills, valleys, and ridges meet each other, and the relative height difference is large. Due to long-term quarrying activities, the ground is seriously damaged, with an area of 10.3 m^2^, of which the bare leakage ground is 5 km^2^, and 1 km^2^ area is a quarry and is being mined.

#### 2.1.2. Technical Planning

For this kind of broken landform, if the traditional manual mapping is used, the field work is very heavy, and there are potential safety hazards in many areas, so that personnel and instruments cannot reach it at all. However, the ordinary aerial survey method can hardly meet the accuracy requirement of 1 : 500 topographic map and cannot meet the requirements of ecological restoration test. Therefore, it is considered to adopt oblique photography technology to obtain a true three-dimensional model with high precision, then draw a large-scale working base map, and obtain the detailed surface geographic information features of this area.

#### 2.1.3. Acquisition of Multiview Oblique Images

According to the geographical conditions and actual requirements of the test area, a small rotary-wing UAV was selected as the flight platform in this test, equipped with five lenses (vertical lens focal length of 35 mm, oblique lens focal length of 50 mm, and pixel size of 4.88 *μ*m) to obtain high-resolution visible light images with multiple perspectives and high overlap at low altitude. To ensure that the image resolution and the reliability of the 3D model can meet the needs of subsequent mine ecological restoration, the preset flight relative altitude is 215 m, the heading overlap is 75%, the distance is 92.1 m, the east-west flight has 32 lanes, the overlap between the lanes is 85%, and the lateral interval is 20 m. The aerial photography resolution is about 3 cm. A total of 19 sorties were planned in the whole shooting area, and 15,860 images were taken, covering an area of about 5.8 km^2^. At the same time, nine image control points were arranged in the test area by GPS ground differential measurement, and the measurement accuracy was better than 0.10 m.

#### 2.1.4. Data Processing Methods

**Figure 1:**
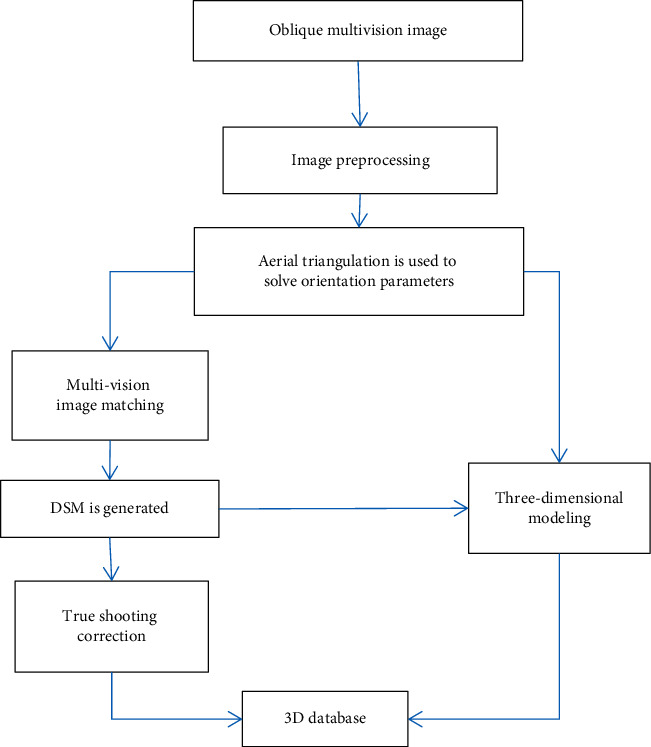
Basic processing flow of tilt photogrammetry.

The aerial photogrammetry system of UAV used in this paper has the characteristics of high resolution, large field of view of image acquisition, and multiple resolutions of images of the same ground object at different acquisition angles. According to these characteristics, data processing needs to go through the technical steps shown in Figure 1.

## 3. Results

### 3.1. Image Data Processing

In this experiment, PhotoMesh software was selected for image processing and modeling, and the connection points of photos were automatically changed according to the way of matching navigation auxiliary data with images. The mismatching points were relatively oriented by the continuous method with model link conditions, and then the triangular grid was constructed. After solving by space three, the initial model was established under the condition that all kinds of tolerances met the specification requirements. After a series of treatments such as stitching and mapping, the true 3D model is constructed. At the same time, it can produce common photogrammetry products such as DOM, DEM, and DLG [9].

#### 3.1.1. Precision Control and Verification

In order to improve the accuracy of the model, 15 photo control points were actually measured in the field, and the control points were turned into the stereo model by means of manual stereo measurement, and the whole adjustment of the regional network was solved. According to statistics, the overall measurement accuracy of image point coordinates is ±1.175 pixels. The data processing and the report of solving space three are shown in Tables 1 and 2.

**Figure 2:**
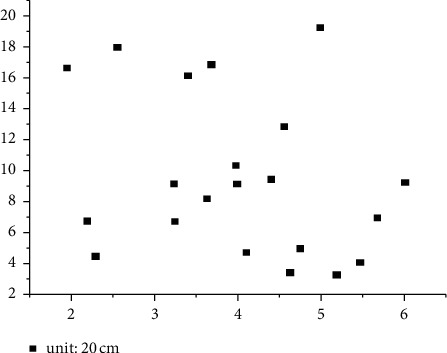
Distribution of checkpoint residuals.

Finally, in order to evaluate the accuracy of the model in this experimental area more objectively, we once again used GPS equipment and uniformly measured 21 detection points in the field as the basis for accuracy verification. The residual distribution is shown in Figure 2.

At the same time, in order to objectively evaluate the accuracy of this 3D model, the plane and elevation maximum residuals of the 5 image control points and 3 inspection points are calculated according to the following formula:

(1) $$\begin{array}{c} 0m_{1}=\pm \sqrt{\overset{n}{\underset{i=1}{\sum }}\frac{\left(\Delta_{i}\Delta_{i}\right)}{n}}. \end{array}$$

The objective function of optimal configuration includes social benefit, economic benefit, and ecological environment benefit. Firstly, the TM (thematic mapper) remote sensing images of the study area are registered and interpreted to obtain the land use types of the study area; then, the water demand of various land use types is calculated with the help of relevant data; and finally, the pixel-based objective function is established. Assuming that there are *I* rows and *J* columns in the decoded image pixels, there are *L* pixels and *K* water industries in total, and the mathematical model for the optimal allocation of multiobjective water resources is as follows:

(2) $$\begin{array}{c} f_{1}\left(x\right)=\max\sum_{k=1}^{K}\left\{\left[e_{k}^{m}-v_{k}^{m}\right]\right\}x_{k}^{m}\alpha_{k}^{m},\\ f_{2}\left(x\right)=\min\sum_{k=1}^{k}\left[D_{k}-\left(x_{k}^{m}+x_{k}^{n}+x_{k}^{p}\right)\right],\\ f_{3}\left(x\right)=\min\sum_{k=1}^{k}\left[0.01\left(x_{k}^{m}+x_{k}^{n}+x_{k}^{p}\right)\sum_{e=1}^{e}C_{k}^{e}O_{k}^{e}\right]. \end{array}$$

In the formula, *f*_1_(*x*) is the economic benefit target, taking the maximum direct economic benefit generated by the water supply pixel as the target; *f*_2_(*x*) is the social benefit target, taking the minimum total water shortage of the pixel as the target; and *f*_3_(*x*) is the goal of ecological and environmental benefits to minimize the emission of important pollutants in pixels. Important pollutants generally include chemical oxygen consumption.

The corresponding composite expression is as follows:

(3) $$\begin{array}{c} C_{m-1},n=\sum_{k}h\left(k-2n\right)C_{m},k, \end{array}$$

(4) $$\begin{array}{c} \sum_{k=1}^{K}\left(x_{k}^{m}+x_{k}^{n}+x_{k}^{9}\right)\leq W, \end{array}$$

(5) $$\begin{array}{c} \sum_{k=1}^{k}x_{k}^{n}\leq W_{\max}^{n}, \end{array}$$

(6) $$\begin{array}{c} \sum_{k=1}^{K}D_{k}\geq \sum_{k=1}^{K}P_{k}S_{k}, \end{array}$$

(7) $$\begin{array}{c} \sum_{k=1}^{K}\sum_{e=1}^{e}\frac{\psi_{k}^{e}D_{k}^{e}}{D_{\max}^{e}}\geq E_{\min}. \end{array}$$

Equation (3) is the constraint of water availability, and the sum of the available water of pixels (*i*, *j*) cannot exceed the average water supply capacity of the water source project to the maximum water supply capacity *W*_max_^*n*^ (unit: m^3^); *W*_max_^*n*^ ≥ 0. Equation (4) is the groundwater exploitation. If groundwater is over exploited, equation (4) is the constraint condition for water use in corresponding industries (domestic, industrial, agricultural, and ecological environment), which represents the standard per capita domestic water consumption, water consumption for 10000 yuan output value, water consumption for farmland irrigation quota, and water consumption for ecological environment in the planning level year; formula (5) is the constraint of the comprehensive evaluation index of water environment; *E*_min_ is the minimum value required by the water environment index; *D*_*k*_^*e*^ and *D*_max_^*e*^, respectively, are the planned horizontal year pixels (*i*, *j*)*k* annual emissions and maximum allowable emissions of pollutants in the industry (unit: t/year); and *ε* is the weight of the pollution factor *ψ*_*k*_^*e*^.

## 4. Analysis

According to the three-level difference results in Table 2, the maximum plane/elevation residual error of the checkpoints measured in the field is 0.221 m−0.181 m, and the median error is 0.030 m−0.112 m. According to China's current aerial photogrammetry specifications and the encryption requirements of 1 : 500 scale topographic mapping in hilly land, the plane position error of inspection points relative to field control points should be less than 0.175 m, and the elevation should be less than 0.280 m. The experimental results can meet the requirements. Combined with the residual distribution diagram shown in Figure 2 of checkpoints, further analysis shows that the plane errors of 21 field survey checkpoints do not show obvious rules in spatial distribution, that is, the way of overall regional network adjustment for multiple navigation belts. The model system error can be effectively eliminated by inclining the model connection conditions with multiple overlaps between photographic bands and a small number of control points in the photographic area, so as to obtain reliable model accuracy [10].

### 4.1. Application of Oblique Photography-Assisted Remote Sensing Technology

Digital achievements such as digital orthophoto map (DOM), digital elevation model (DEM), digital line drawing (DLG), and true three-dimensional model (3D-model) with high precision are generated by means of oblique photography technology, which quickly provides accurate and reliable basic data, working base map, and objective data basis for mine ecological restoration planning, design, construction and monitoring, and evaluation of restoration achievements.

### 4.2. Ecological Restoration Planning

Photographs taken by aircraft cannot be affected by topography, especially the five-spelling tilt camera, which collects the scene images from five different angles at the same time, observes and browses at different angles and scales through operations such as zooming in, zooming out, rotating, and pitching, and comprehensively and thoroughly grasps the scene surface conditions and overcomes the “blind zone” caused by the steep terrain and inaccessible manpower. Due to the high accuracy of the model, the actual damage degree can be measured more accurately by measuring, calculating, and cutting the section view in any direction directly on the model, so that most of the field survey and measurement work is transferred to the collection work of the internal model. On the premise of ensuring the quality and accuracy of the results, it greatly reduces the field workload, improves the efficiency, and reduces the operating cost. Scheme design based on three-dimensional model can simulate, display, and compare the effects of various proposed schemes and then continuously adjust and select the best ones. Through the superposition and comparison of the effects of several schemes, the optimal scheme can be easily selected to avoid mistakes caused by human factors. The restoration works in this test area mainly include dangerous rock cleaning, slope protection, construction of intercepting and drainage ditch and retaining wall, soil covering and transportation, and green planting and sowing. In the ecological restoration construction, the excavation quantity, filling quantity, and filling height can be accurately calculated, which makes the construction operation more accurate and plays a guiding role. Ecological restoration construction can also make use of the height correspondence between the model and the field, which makes a lot of construction operations into construction lofting, which is more convenient and easier to control the quality. Through aerial photography for many times, the image data of each stage before and after the restoration and during the restoration process were collected. Through on-site real-life comparison and data analysis and monitoring, the land surface change, slope and retaining wall stability, vegetation survival rate, and growth were fully grasped. Give an objective assessment of hazard sources and ecological restoration effect, so as to find problems in time and give reasonable treatment measures in time.

## 5. Conclusion

Oblique photography technology can provide more abundant digital achievements, which can not only assist remote sensing technology but also play an important role in restoration scheme design, restoration construction, and monitoring and management after restoration. It quickly provides accurate and reliable basic data, working base map and objective data basis for the planning, design and construction of mine ecological restoration, and the monitoring and evaluation of restoration results and gradually realizes the virtuous circle of mining damage control.

## Figures and Tables

**Table 1:** Basic information of data processing.

Number of photos: 15590	Aerial photo area: 5.0738 km^2^
Spatial reference: Xi'an 1980 coordinate system is zoned at 3 degrees, and the central meridian is 111	Elevation datum: geoid height
Control points: 9	Check points: 6
Connection points: 3.58424 million	Treatment time: 20 d19h

**Table 2:** Processing accuracy of empty three.

Maximum residual error of control point/m		Error in control residual error/m	
Plane	Elevation	Plane	Elevation
0.106	0.112	0.077	0.740
Maximum value of checkpoint residual error/m		Error in control residual error/m	
Plane	Elevation	Plane	Elevation
0.221	0.181	0.030	0.112

## Data Availability

The data used to support the findings of this study are available from the corresponding author upon request.
